# Fair AI-powered orthopedic image segmentation: addressing bias and promoting equitable healthcare

**DOI:** 10.1038/s41598-024-66873-6

**Published:** 2024-07-12

**Authors:** Ismaeel A. Siddiqui, Nickolas Littlefield, Luke A. Carlson, Matthew Gong, Avani Chhabra, Zoe Menezes, George M. Mastorakos, Sakshi Mehul Thakar, Mehrnaz Abedian, Ines Lohse, Kurt R. Weiss, Johannes F. Plate, Hamidreza Moradi, Soheyla Amirian, Ahmad P. Tafti

**Affiliations:** 1 Department of Health Information Management, University of Pittsburgh, Pittsburgh, 15620 USA; 2 Intelligent Systems Program, University of Pittsburgh, Pittsburgh, 5620 USA; 3 Department of Orthopaedic Surgery, University of Pittsburgh, Pittsburgh, 15213 USA; 4 Cortechs.ai, San Diego, 92122 USA; 5 Department of Computer Science, North Carolina Agricultural and Technical State University, Greensboro, 27411 USA; 6 Seidenberg School of Computer Science and Information Systems, Pace University, New York, 10038 USA

**Keywords:** Medical imaging, Musculoskeletal system

## Abstract

AI-powered segmentation of hip and knee bony anatomy has revolutionized orthopedics, transforming pre-operative planning and post-operative assessment. Despite the remarkable advancements in AI algorithms for medical imaging, the potential for biases inherent within these models remains largely unexplored. This study tackles these concerns by thoroughly re-examining AI-driven segmentation for hip and knee bony anatomy. While advanced imaging modalities like CT and MRI offer comprehensive views, plain radiographs (X-rays) predominate the standard initial clinical assessment due to their widespread availability, low cost, and rapid acquisition. Hence, we focused on plain radiographs to ensure the utilization of our contribution in diverse healthcare settings, including those with limited access to advanced imaging technologies. This work provides insights into the underlying causes of biases in AI-based knee and hip image segmentation through an extensive evaluation, presenting targeted mitigation strategies to alleviate biases related to sex, race, and age, using an automatic segmentation that is fair, impartial, and safe in the context of AI. Our contribution can enhance inclusivity, ethical practices, equity, and an unbiased healthcare environment with advanced clinical outcomes, aiding decision-making and osteoarthritis research. Furthermore, we have made all the codes and datasets publicly and freely accessible to promote open scientific research.

## Medical image analysis in orthopedics: fair and unbiased AI-powered segmentation

Diagnostic imaging has been an integral component of clinical care for over a century. By obtaining images through different imaging modalities (e.g., X-ray, CT, MRI), healthcare providers gain credible insights about patient health, clinical outcomes, and treatment needs, with plain radiographs (X-rays) playing a critical role in initial assessments and ongoing monitoring, especially in resource-limited settings. While CT and MRI imaging provide more detailed insights, X-ray imaging is routinely prioritized first due to its accessibility and cost-effectiveness^1,2^. In orthopedics and rheumatology, plain radiographs are used to identify several diseases and complications, including broken bones, osteoarthritis, osteoporosis, rheumatoid arthritis, torn ligaments, lucent bone lesions, and joint loosening^3^. To further streamline radiograph interpretation, medical image segmentation is a critical process that identifies and delineates specific structures or regions of interest (ROIs) within diagnostic images^4^. Segmentation characterizes diagnostic images in clinical settings by their most relevant components, such as anatomical structures, organs, bones, and tissues. It addresses variable contrast and noise within patient radiographs, which often obscure the ROIs. By precisely outlining the ROIs in an interpretable manner, medical image segmentation is a powerful tool for pre-operative planning and post-operative assessments. Specifically, in the context of hip and knee bony anatomy, comparing segmentation masks of radiographs between patients can provide insights into pain progression or the need for total joint arthroplasty^5,6^.

Recent advancements in computer vision algorithms empowered by deep learning have demonstrated remarkable success in medical image segmentation^7–12^. These techniques have significantly improved the efficiency and accuracy of segmentation tasks. However, concerns regarding fairness and potential biases inherent in AI-powered models remain^13–19^. Biases in medical image segmentation refer to systematic errors or inconsistencies in the algorithm’s predictions that may disproportionately impact specific groups of patients. Addressing these biases is crucial for ensuring equitable and accurate segmentation results, which maximizes patient safety across diverse populations^16,20–22^. Minimizing such bias entails that a deep learning/AI algorithm will perform equitably across various patient populations, becoming a fair AI model. Fair image segmentation thus has the potential to mitigate inherent biases and disparities, which can profoundly impact patient care and treatment plans. Knee and hip image segmentation are integral to orthopedic surgery, playing a crucial role in preoperative planning and postoperative assessments^23–26^. Fairness in these segmentation methods is especially critical when surgeons consider anatomical differences between patients, as these differences can significantly impact surgical outcomes and treatment plans. The necessity for fair segmentation methods arises from the variability in anatomy, joint structures, and bone densities across diverse demographic groups, including sex, race, and age. For example, female pelvises are ubiquitously wider than male pelvises, an evolutionary adaptation for childbirth^27^. Similarly, racial disparities in bone density persist even after adjustments for anthropometric, lifestyle, and biochemical factors, with African Americans exhibiting higher bone density levels compared to their White counterparts^28^. Age-related changes, such as the decline in bone mineral density, also vary significantly and are crucial in diagnosing and treating conditions like osteoporosis^29^.

This study aims to revisit contemporary advancements in deep learning-based segmentation of knee and hip bony anatomy, focusing on mitigating potential biases related to sex, race, and age in the segmentation of bony anatomy using plain radiographs. Our approach addresses the critical need for fairness and accuracy in medical image analysis, recognizing the significant impact of bias on patient care and treatment outcomes. Our proposed framework for the AI-driven method is composed of three key components: data pre-processing, the creation of a gold-standard image segmentation dataset, and the development of a fair hybrid AI model. We assembled a gold-standard dataset using reliable data sources, comprehensive annotation guidelines, trained annotators, and rigorous annotation evaluation. Our hybrid AI model was trained with four distinct bias mitigation strategies to ensure fairness and accuracy in knee and hip image segmentation. These strategies were implemented to address and minimize potential biases, thereby enhancing the accuracy and reliability of the segmentation results. To the best of our knowledge, this study represents the first initiative to build, train, and validate deep learning-enabled knee and hip bony anatomy segmentation methods that generate accurate and unbiased results across diverse sex, race, and age. Our comprehensive approach advances the state of the art in medical image segmentation and contributes to the broader goal of promoting fairness in AI-driven healthcare solutions.

## Novelties of this work

This study focuses on advancing fair and unbiased knee and hip image segmentation autonomously with a dual commitment to technical innovation and clinical impact as follows:

### Technical novelties

The three-fold technical novelties of this work include:

1. Automated knee/hip bony anatomy segmentation system: We introduce an automated image segmentation system tailored for knee and hip bony anatomy segmentation using plain radiographs, showcasing technical prowess in algorithmic design and implementation. The key novelty lies in the replacement of the down-sampling encoder in U-Net with ResNet18 and EfficientNet-B0, resulting in a synergistic fusion that amplifies the capabilities of both deep learning models. The framework of this procedure is illustrated in Fig. 1.

**Figure 1:**
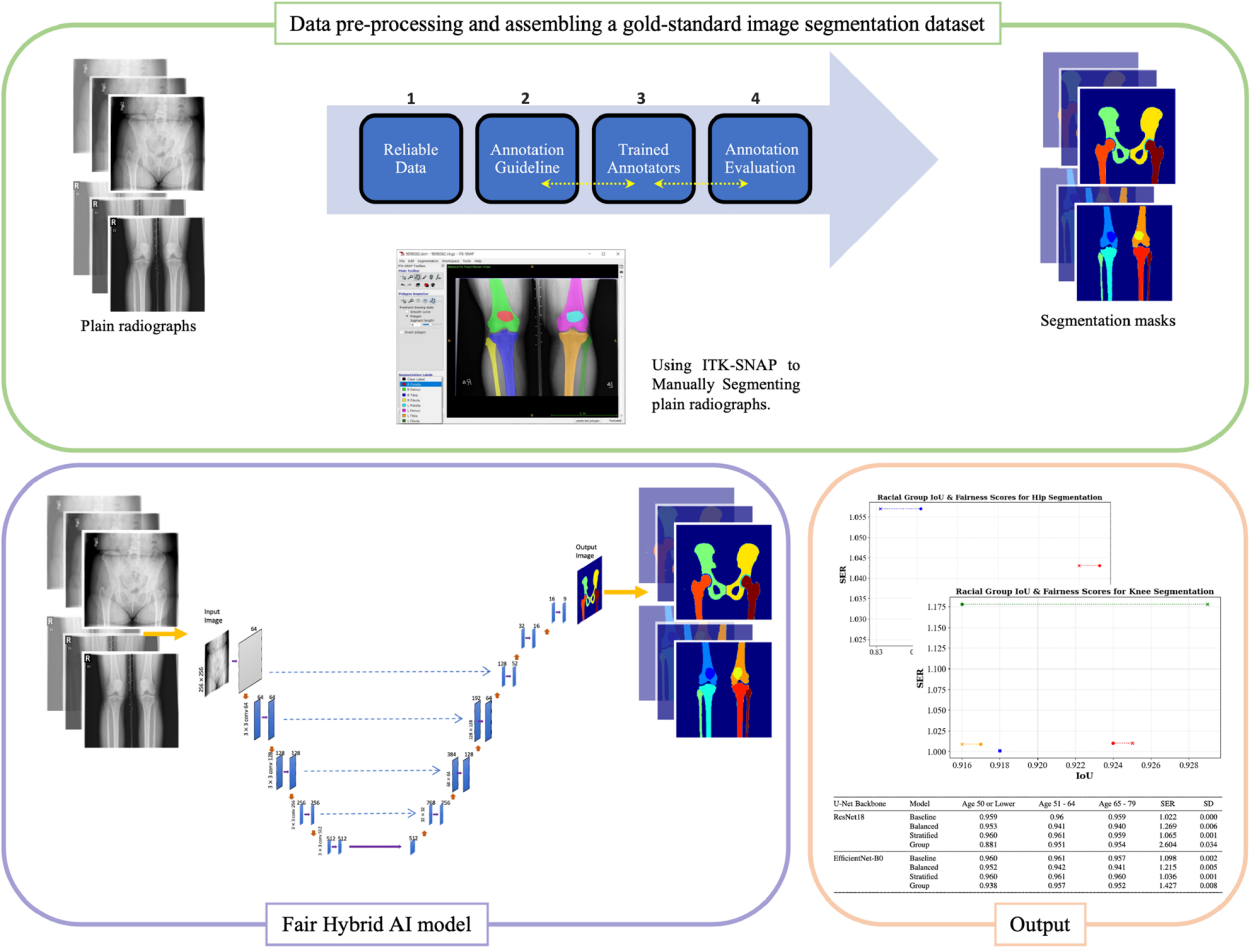
The proposed framework of our AI-driven method, depicting the data pre-processing steps, the creation of a gold-standard image segmentation dataset, and the integration of our Fair Hybrid AI model.
2. Bias mitigation in knee/hip bony anatomy segmentation: We propose computational strategies to ensure fair and unbiased image segmentation, elevating the technical standards in the field of knee/hip bony anatomy segmentation.
3. Open scientific research: Simultaneously, we have made all the codes and datasets publicly and freely available, fostering collaborative research, education, and benchmarking within our scientific community.

### **Clinical novelties**

The clinical novelties of this work lie in three-fold:

1. Equitable healthcare provision: Our knee/hip bony anatomy segmentation system enhances equitable healthcare by providing accurate diagnoses and treatment for diverse patient populations, leading to improved clinical outcomes and a more inclusive healthcare landscape.
2. Pre-operative planning advancements: Unbiased image segmentation provides surgeons with precise, bias-free insights into a patient’s bone anatomy, enabling tailored pre-operative planning to assess crucial deformities and variations. This approach reduces the need for revision surgeries, optimizing healthcare resources and improving patient and clinical outcomes.
3. Customized surgical plans: Unbiased bone anatomy segmentation aids orthopedic surgeons in creating personalized surgical plans, optimizing surgical strategies and implant selection for improved postoperative outcomes and long-term success in Knee Arthroplasty (TKA)/Total Hip Arthroplasty (THA) procedures.

## Materials and methods

The proposed framework of our AI-driven method is illustrated in Fig. 1. It includes data pre-processing, establishing a gold-standard image segmentation dataset, and the proposed fair hybrid AI model, respectively.

### Data and data-preprocessing

**Figure 2:**
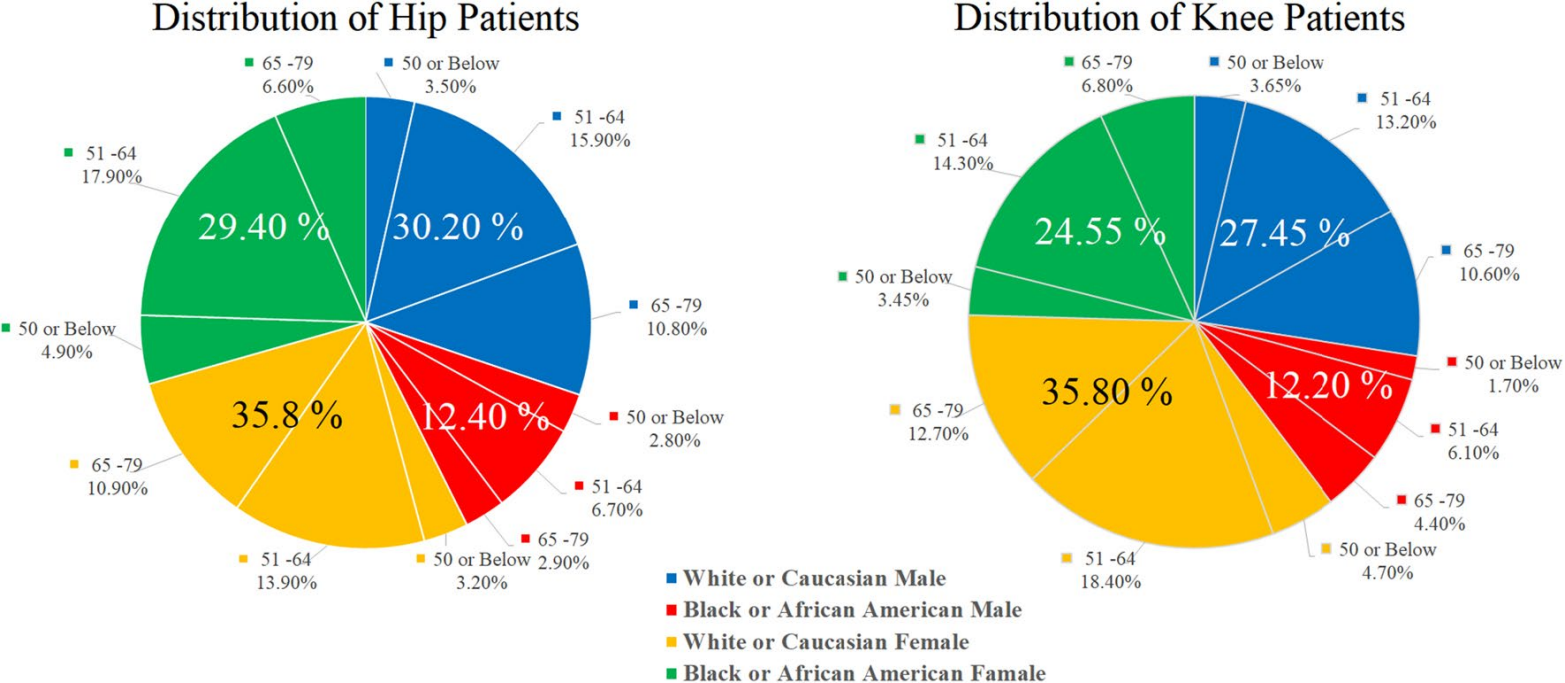
Distribution of Hip and Knee Patients concerning sex, race, and age. Black or African American Males have the lowest representation within the dataset, accounting for only 12.4% of Hip patients and 12.2% of Knee patients. White Males and Females comprise the majority of the dataset, accounting for a combined 66% percent of Hip patients, and 63.25% of Knee patients.

We computationally assembled a retrospective cohort from the Osteoarthritis Initiative (OAI)^30^. Our cohort included 766 patients for the hip and 707 patients for the knee, with one hip or knee radiograph per patient. This resulted in a total of 1473 plain radiographs. Sex, race, and age group distribution within our dataset is demonstrated in Table 1. Figure 2 presents a pie chart depicting the distribution of hip and knee patients, regarding different sex, race, and age groups.

**Table 1:** Sex, racial, and age group distribution within our annotated dataset.

Sex	Race	Age group	Hip		Knee	
			Patients	Percentage	Patients	Percentage
Male	White or Caucasian	50 or Below	27	3.5	26	3.65
		51–64	121	15.9	93	13.2
		65–79	82	10.8	75	10.6
	Black or African American	50 or Below	21	2.8	12	1.7
		51–64	51	6.7	43	6.1
		65–79	22	2.9	31	4.4
Female	White or Caucasian	50 or Below	24	3.2	33	4.7
		51–64	106	13.9	130	18.4
		65–79	83	10.9	90	12.7
	Black or African American	50 or Below	38	4.9	25	3.45
		51–64	136	17.9	101	14.3
		65–79	50	6.6	48	6.8

### Gold-standard imaging dataset for image segmentation

The establishment of a gold-standard imaging dataset is a critical prerequisite for building clinically applicable deep learning models in the field of medical imaging. Our proposed pipeline to assemble a gold-standard imaging dataset is demonstrated in the first part of Fig. 1, entitled *Data pre-processing and assembling a gold-standard image segmentation dataset*. The pipeline includes the following steps: (1) Reliable Data: Patient radiographs were acquired from a reputable source, the Osteoarthritis Initiative (OAI)^30^ dataset and it abides by standard image formats, the DICOM (Digital Imaging and Communications in Medicine) images. (2) Annotation Guideline: Annotation guidelines were developed by orthopedic surgeons, where the guidelines provide clear instructions on annotating medical images with high reliability and precision. (3) Trained Annotators: The guidelines assisted annotators with medical image segmentation software application, the ITK-SNAP^31^. The iterative nature of our group’s annotation process became apparent as we initiated the first rounds of assessing inter-rater agreements among our annotators. In response to the initial findings, we found it necessary to make slight revisions to the annotation guidelines, aiming to improve inter-rater agreement. The entire process was conducted under the supervision of an orthopedic surgeon, ensuring that the adjustments made were not only effective but also aligned with the clinical expertise required for accurate medical image annotation. (4) Annotation Evaluation: Tagging evaluation is the process of assessing the accuracy and quality of tagging data. Concerning segmentation, the tagging data are the regions of interest and the category of bony anatomies, such as patella, femur, and tibia for plain knee radiographs.

**Figure 3:**
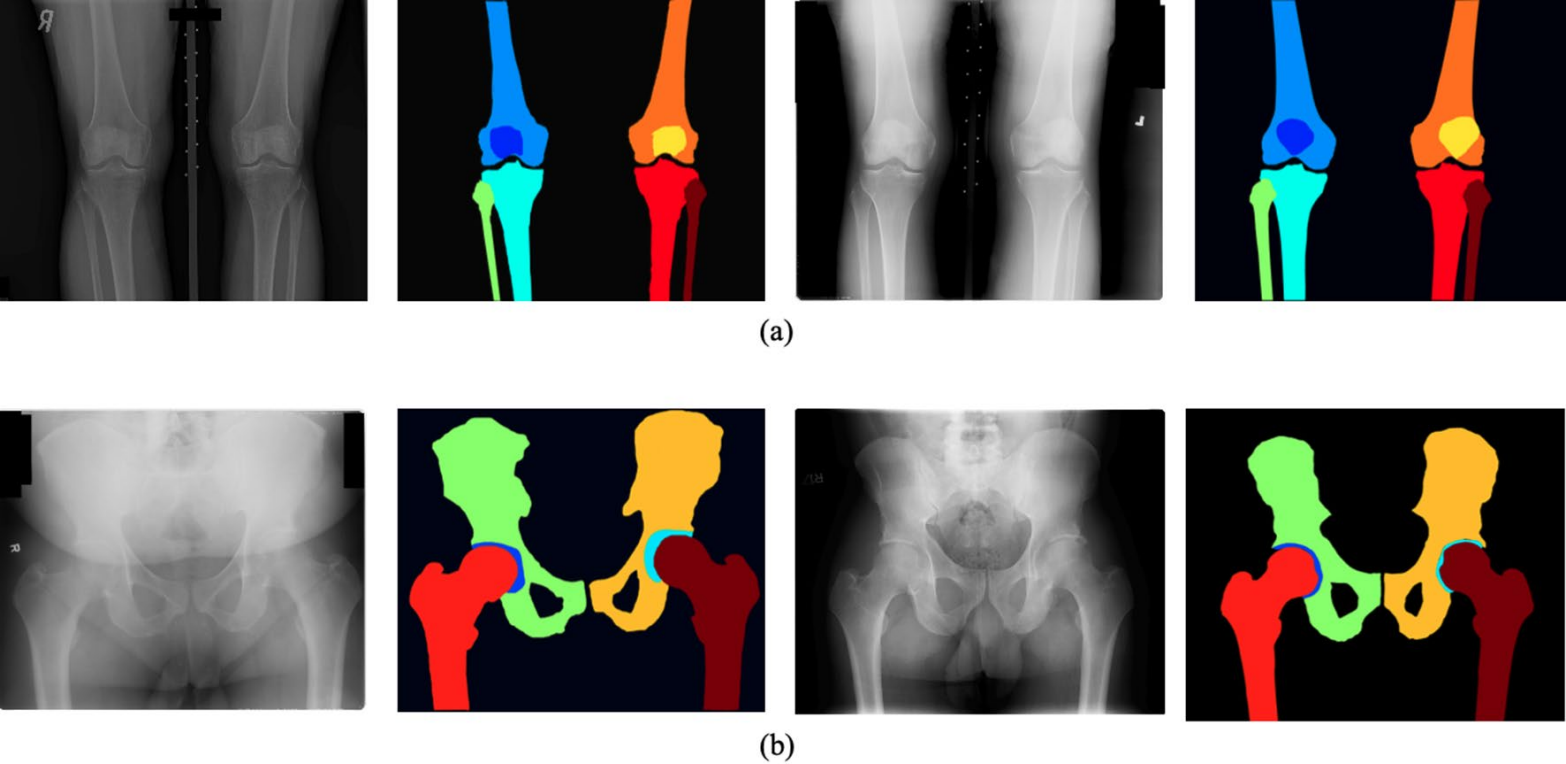
(**a**) Plain knee radiographs along with their manual segmentation masks. (**b**) Plain hip radiographs along with their manual segmentation masks.

We utilized Intersection over Union (IoU) to ensure a stable and promising inter-rater agreement among our annotators, where the average IoU among the annotators was 0.873 for the knee and 0.830 for the hip. An example of a manual segmentation task by ITK-SNAP is illustrated in Fig. 1. Figure 3 also shows a few examples of plain knee and hip radiographs along with their manual segmentation masks.

### Quantifying image segmentation accuracy with IoU and dice scores

Image segmentation could be considered as a pixel-wise classification problem. The most commonly known performance metrics for any AI-enabled classification task include precision, accuracy, recall, and F1 score. However, in the context of image segmentation, the Intersection over Union (IoU) and Dice Score offer further information about the discrepancies between the segmentation masks predicted by the AI model and the ones provided manually by the image annotators as gold-standard masks.

IoU provides valuable insights into the concordance between two sets of tensors that depict the gold-standard (ground-truth) and the predicted segmentation, and it quantifies the extent of overlap between two sets of pixels contained within segmentation masks. It computes the ratio of the pixel intersection to the pixel union between the predicted segmentation masks (*predicted*) and the gold-standard masks (*actual*) presented in Eq. (1):

1 $$  \begin{aligned} IoU = \frac{\text {Intersection}_{ predicted\,\, \& \,\, actual}}{\text {Union}_{ predicted \,\, \&  \,\,actual}} \end{aligned}$$

here, the intersection refers to the pixels that are correctly classified as positive in both the predicted and gold-standard segmentation. The union represents the total number of pixels that are classified as positive in either of the two segmentation masks. The IoU value ranges from 0 to 1, where a higher value indicates a greater spatial overlap and also a higher agreement between the annotations. A value of 1 signifies perfect agreement, while values between 70 and 90% demonstrate a high degree of overlap between the predicted and gold-standard segmentation masks.

Similar to IoU, the Dice Score provides valuable insights into the concordance between two sets of tensors that depict the gold-standard (ground-truth) and the predicted segmentation by evaluating the similarity of the segmented regions of the prediction and ground-truth. It computes the ratio of 2 times the pixel intersection to the pixel union between the predicted segmentation masks (*predicted*) and the gold-standard masks (*actual*) presented in Eq. (2):

2 $$  \begin{aligned} Dice = \frac{2 \times \text {Intersection}_{ predicted \,\, \& \,\, actual}}{\text {Union}_{ predicted \,\, \&  \,\,actual}} \end{aligned}$$

here, the intersection refers to twice the number of pixels correctly classified as positive in both the predicted and ground truth segmentation, giving a higher significance to the correctly classified regions. Like the IoU score, the dice score value ranges from 0 to 1, where a higher value indicates a greater agreement and accuracy between the annotations. A value of 1 signifies perfect agreement, while values between 70 and 90% demonstrate a high degree of overlap and agreement between the predicted and gold-standard segmentation masks.

### Fair hybrid AI model for image segmentation

Over the past decade, deep learning models have emerged as the dominant methodologies for achieving remarkable performance in image segmentation tasks. In the context of medical image segmentation, one particular model, known as U-Net^32^, has already demonstrated very successful application in several medical imaging informatics settings, such as brain images^33,34^, liver images^35,36^, and hip and knee images^37–40^. Its popularity can be attributed to its exceptional proficiency in capturing intricate image details and spatial data patterns. U-Net offers a unique architectural design, comprising both an encoder and decoder framework, coupled with skip connections. In the course of this study, we have harnessed the power of U-Net, enhanced with two different backbones: ResNet18^41^ and EfficientNet-B0. These hybrid models are deployed to perform the challenging task of segmenting hip and knee bony anatomy in medical images. The decision to enhance U-Net with a ResNet18 and EfficientNetB0 backbone was made to leverage the strengths of both architectures, combining U-Net’s robust segmentation capabilities with ResNet18’s and EfficientNet’s deep feature extraction capabilities. However, due to the similar capabilities and the results of both models, in the end, we stick with ResNet18. The ResNet18 backbone consists of 4 encoder layers that sequentially increase the number of feature maps from 64 to 128 to 256 and finally to 512. For each layer, two encoder blocks utilize $3 \times 3$ convolutional filters, a stride of 2, and a padding of size 1. The output of each layer is calculated as Eq. (3):

3 $$\begin{aligned} L_i = E_{i, 2}(E_{i, 1}(L_{i-1})) \end{aligned}$$

where $L_i$ is the output for the corresponding layer in the backbone and $E_{i,1}$ and $E_{i, 2}$ correspond to the two encoders for the layer and $L_{i-1}$ is the output of the previous layer. The first block of each layer also consists of a downsampling which is then combined with the corresponding layer of the decoder as follows (Eq. 4):

4 $$\begin{aligned} D_{i, 1} = Concat(E_{j, 1} + D_{i-1, 2}) \end{aligned}$$

where $D_{i,1}$ is the first block in decoder *i*, $D_{i-1, 2}$ is the output of the second block of the previous decoder, and $E_{j, 1}$ is the first encoder block of the corresponding layer of the encoder with the same output size as the input of the $D_{i, 1}$. This is only done for the first 4 decoder blocks. This fusion of cutting-edge deep learning techniques aims to provide more accurate and reliable results in the field of medical image analysis, with potential implications for improved diagnosis and treatment planning in orthopedic medicine.

Our hybrid AI model was then equipped with four different bias mitigation strategies to computationally tackle the problem of biased image segmentation. Therefore, we undertook a comprehensive approach by conducting multiple model training experiments as follows:

- Baseline model. Due to the potential for bias in deep learning models, we aimed to establish a baseline model for comparison before exploring different bias mitigation techniques. This model was trained on the entire training dataset (combined groups), ensuring that the distribution of the protected attributes, such as sex and race, accurately represent the entire dataset. We refer to this model hereafter as the *baseline model*.
- Balanced dataset model. To address potential bias and improve the model’s ability to accurately classify different examples from the underrepresented groups, we utilized under-sampling of the overrepresented protected groups in the training data. This approach aimed to mitigate the risk of the model exhibiting bias towards the majority groups and improve its ability to accurately classify the minority groups. We refer to this model hereafter as the *balanced model*.
- Stratified batching model. To ensure that protected groups of interest have a balanced representation during training we utilize stratified batching. This approach groups examples based on the protected attributes and then randomly samples instances from each group. This creates mini-batches for training that have an unbiased distribution of examples for each protected group in every training batch. This helps in promoting fairness during the learning process. We refer to this model hereafter as the *stratified model*.
- Group-specific model. To enhance performance for minority groups we explore training individual models that are tailored for each group (e.g., female or male). This allowed for the evaluation of the effectiveness of group-specific models in mitigating bias and improving the overall fairness of the models. We refer to this model hereafter as the *group-specific model*.

### AI fairness evaluation metric

We evaluated the effectiveness of various bias mitigation techniques by measuring the models’ performance and contrasting the outcomes with those of the baseline model. This comprehensive approach enabled us to gauge the influence of these techniques and assess their efficacy in promoting fairness and mitigating bias within the models. To evaluate the fairness of our models, we utilized the well-established Skewed Error Ratio (SER), focusing specifically on evaluating potential biases in the algorithm’s prediction errors toward specific groups. SER, shown in Eq. (5), utilizes the ratio of highest to lowest error rate among protected groups, with *g* denoting the considered protected groups. It should be noted that higher values of SER present a higher presence of bias, while values closer to one are an indication of a lower presence of bias. Utilizing this metric offers valuable perspectives into the dispersion of IoU values among diverse racial and sex groups, assisting in the detection of discrepancies and imbalances.

We have also incorporated the Standard Deviation (SD). SD quantifies the spread of errors from the mean value. By assessing the SD of the errors, we can gain an understanding of how the IoU values are scattered and deviated from the average. This, in return, allows us to explore the error variation within and among protected groups.

5 $$\begin{aligned} SER_{g}= \frac{Max_g (1 - IoU_g)}{Min_g ( 1- IoU_g)} \end{aligned}$$

## Experimental validation and results

To evaluate the performance of our fair hybrid AI model(s) for hip and knee bony anatomy segmentation, we conducted different experiments by sampling the training data with consideration of the protected groups. This, in return, resulted in different training dataset arrangements, with a focus on *race* and *sex*.

### Experimental setup and model training

To run our experiments, we utilized Jupyter Notebook sessions using the Data Science service on Oracle Cloud that was equipped with a Nvidia V100 GPU with 16GB of memory. For training, validation, and testing of our models, we utilized Python 3.10.11 and PyTorch 1.13.1. When generating the dataset for training, validation, and testing, then 70% of the data was used for training, 15% was used for validation, and 15% was used for testing. To ensure we achieved high segmentation accuracy, we conducted multiple experiments to identify the optimal set of hyper-parameters, specifically focusing on the learning rate and loss functions. We tried different combinations of learning rates (5e−03, 5e−04, and 5e−05) and loss functions (Cross-Entropy loss, Jaccard loss, and Dice loss). All experiments were trained for 50 epochs with a batch size of 16. Ultimately, the best model utilized an Adam optimizer with a learning rate of 5e−04 and Cross Entropy loss. To gauge the models’ accuracy throughout our experiments we utilized IoU and Dice scores with a threshold of 0.5.

### Qualitative and quantitative evaluation

Table 1 and Fig. 2 demonstrate the distribution of the data across different sex, race, and age groups for both hip and knee patients. It reveals that the sample predominantly consisted of white or Caucasian female participants, accounting for 35.8% of the total for both the hip and 61% for the knee with a majority between the age of 51–64. Along with this, it also indicates that overall White/Caucasians constituted a greater percentage of participants than Black/African Americans, with Black/African Americans constituting 42.6% of the total for the hip and 37.65% for the knee.

The IoU scores for hip segmentation across different racial groups are displayed in Table 2a. For ResNet18, the baseline model shows high IoU scores (0.876 for White/Caucasian individuals and 0.867 for Black/African American individuals), but applying group-specific models to mitigate racial biases reduces these to 0.854 and 0.851, respectively, while increasing fairness. The EfficientNet-B0 baseline achieves slightly lower IoU scores (0.869 and 0.862), with the balanced models improving fairness (SER of 1.013) but reducing IoU. The stratified model for ResNet18 and the group-specific model for EfficientNet-B0 exhibit the worst fairness (SER values of 1.047 and 1.076).

**Table 2:** IoU and fairness scores for hip segmentation across different protected attributes, including race, sex, and age.

U-Net backbone	Model	White/Caucasian	Black/AA*	SER	SD	
(a) Racial group IoU scores & fairness scores						
ResNet18	Baseline	0.876	0.867	1.070	0.004	
	Balanced	0.857	0.853	1.027	0.002	
	Stratified	0.864	0.857	1.054	0.004	
	Group	0.854	0.851	1.019	0.001	
EfficientNet-B0	Baseline	0.869	0.862	1.051	0.003	
	Balanced	0.842	0.844	1.013	0.001	
	Stratified	0.876	0.868	1.064	0.004	
	Group	0.861	0.851	1.076	0.005	

U-Net Backbone	Model	Male	Female	SER	SD	
(b) Sex group IoU & fairness scores						
ResNet18	Baseline	0.874	0.871	1.023	0.001	
	Balanced	0.855	0.854	1.010	0.001	
	Stratified	0.873	0.867	1.047	0.003	
	Group	0.851	0.848	1.020	0.001	
EfficientNet-B0	Baseline	0.869	0.864	1.033	0.002	
	Balanced	0.851	0.828	1.155	0.012	
	Stratified	0.870	0.864	1.046	0.003	
	Group	0.843	0.858	1.101	0.007	

(c) Age group IoU & fairness scores						
U-Net Backbone	Model	Age 50 or Lower	Age 51–64	Age 65–79	SER	SD
ResNet18	Baseline	0.868	0.874	0.871	1.044	0.002
	Balanced	0.855	0.833	0.834	1.147	0.010
	Stratified	0.867	0.868	0.862	1.050	0.003
	Group	0.768	0.852	0.852	1.574	0.040
EfficientNet-B0	Baseline	0.866	0.868	0.864	1.025	0.001
	Balanced	0.833	0.817	0.814	1.115	0.008
	Stratified	0.869	0.872	0.868	1.033	0.002
	Group	0.743	0.862	0.843	1.854	0.052

*AA: African American.

**Figure 4:**
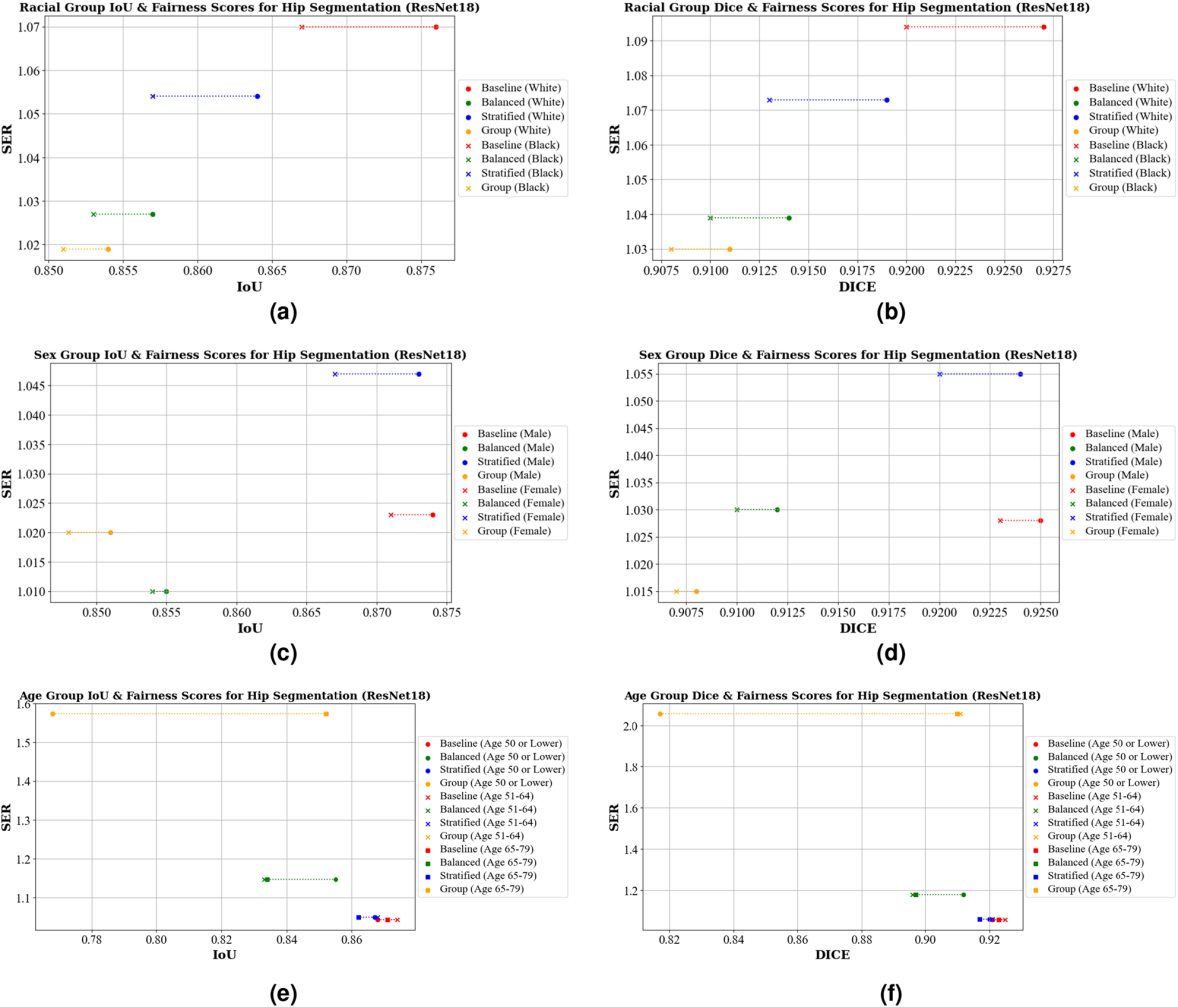
IoU, Dice, and Fairness Scores for hip segmentation using a U-Net model with a ResNet18 backbone. Diagrams (**a**) and (**b**) present the IoU, Dice, and Fairness scores for racial groups. Diagrams (**c**) and (**d**) show the IoU, Dice, and Fairness scores for sex groups. Diagrams (**e**) and (**f**) depict the IoU, Dice, and Fairness scores for age groups.

**Figure 5:**
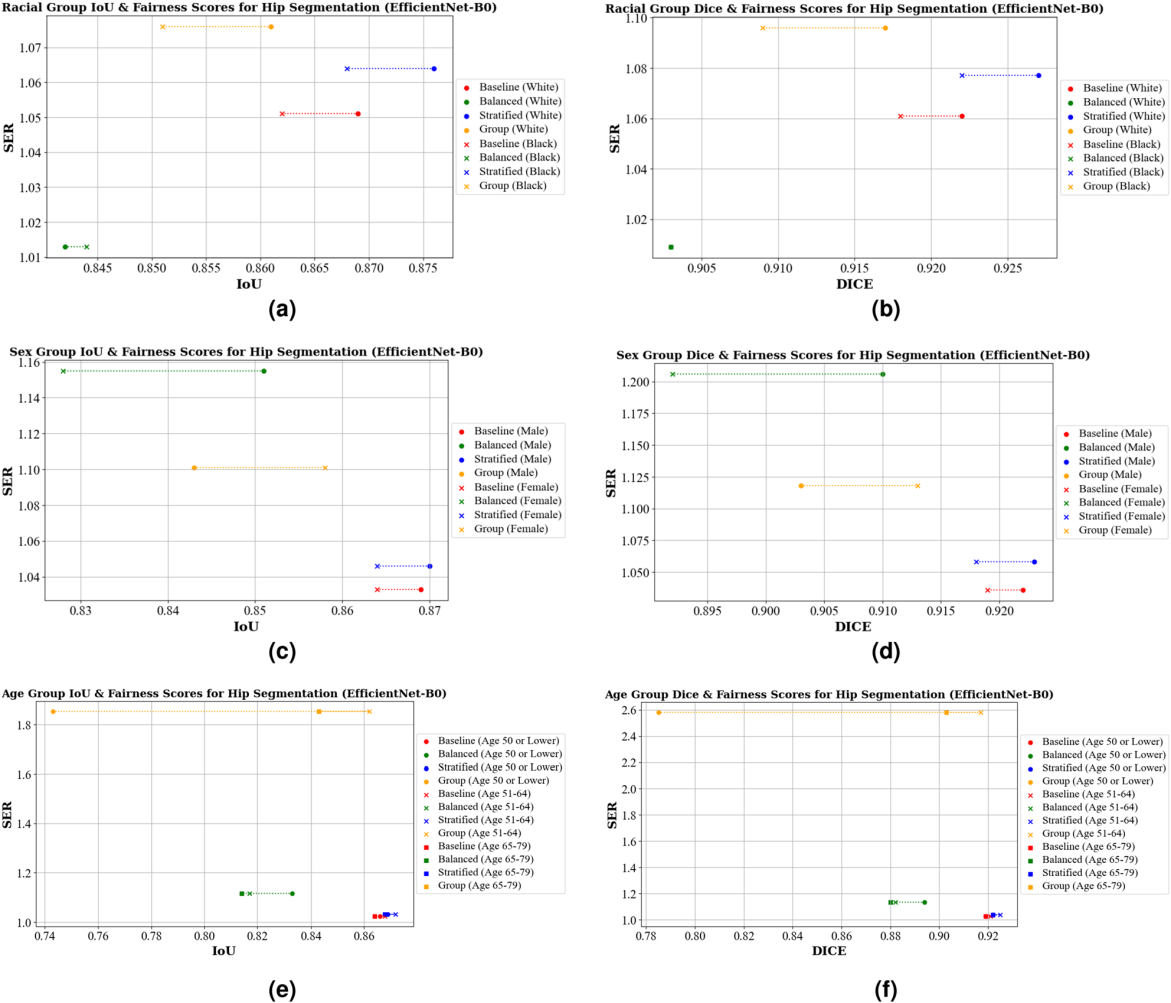
IoU, Dice, and Fairness Scores for hip segmentation using a U-Net model with an EfficientNet-B0 backbone. Diagrams (**a**) and (**b**) present the IoU, Dice, and Fairness scores for racial groups. Diagrams (**c**) and (**d**) show the IoU, Dice, and Fairness scores for sex groups. Diagrams (**e**) and (**f**) depict the IoU, Dice, and Fairness scores for age groups.

Comparatively, in Table 3a the Dice score across racial groups are displayed. The ResNet18 baseline performs well (0.927 for White/Caucasian and 0.920 for Black/African American individuals) but has the highest bias (SER of 1.094). Group-specific models improve the fairness (SER of 1.030) but do so with a decrease in overall performance. EfficientNet-B0 achieves lower baseline scores (0.922 and 0.918). The balanced model was the most fair (SER value of 1.009) indicating almost perfect fairness between groups, but with lower scores (0.912 and 0.910). The graphs in Figs. 4a,b and 5a,b visually depict these trade-offs, highlighting that models with higher fairness are positioned lower on the graph, while those with higher average accuracy (IoU) are on the right, illustrating the bias in model predictions.

**Table 3:** Dice score and fairness scores for hip segmentation across different protected attributes, including race, sex, and age.

U-Net Backbone	Model	White/Caucasian	Black/AA*	SER	SD	
(a) Racial group dice scores & fairness scores						
ResNet18	Baseline	0.927	0.920	1.094	0.003	
	Balanced	0.914	0.910	1.039	0.002	
	Stratified	0.919	0.913	1.073	0.003	
	Group	0.911	0.908	1.030	0.001	
EfficientNet-B0	Baseline	0.922	0.918	1.061	0.002	
	Balanced	0.903	0.903	1.009	0.000	
	Stratified	0.927	0.922	1.077	0.003	
	Group	0.917	0.909	1.096	0.004	

U-Net Backbone	Model	Male	Female	SER	SD	
(b) Sex group dice scores & fairness scores						
ResNet18	Baseline	0.925	0.923	1.028	0.001	
	Balanced	0.912	0.910	1.030	0.001	
	Stratified	0.924	0.920	1.055	0.002	
	Group	0.908	0.907	1.015	0.001	
EfficientNet-B0	Baseline	0.922	0.919	1.036	0.001	
	Balanced	0.910	0.892	1.206	0.009	
	Stratified	0.923	0.918	1.058	0.002	
	Group	0.903	0.913	1.118	0.005	

U-Net Backbone	Model	Age 50 or Lower	Age 51–64	Age 65–79	SER	SD
(c) Age group dice score & fairness scores						
ResNet18	Baseline	0.921	0.925	0.923	1.058	0.002
	Balanced	0.912	0.896	0.897	1.180	0.007
	Stratified	0.920	0.921	0.917	1.059	0.002
	Group	0.817	0.911	0.910	2.049	0.044
EfficientNet-B0	Baseline	0.919	0.921	0.919	1.028	0.001
	Balanced	0.894	0.882	0.880	1.136	0.006
	Stratified	0.922	0.925	0.922	1.039	0.001
	Group	0.785	0.917	0.903	2.581	0.059

*AA: African American.

In Table 2b we present the results for hip segmentation across different sex groups. For IoU scores, the ResNet18 baseline achieves 0.874 for males and 0.871 for females, while EfficientNet-B0 shows slightly lower scores with 0.869 for males and 0.864 for females. The group-specific model shows the highest fairness (SER of 1.019), while the balanced model for EfficientNet-B0 is the fairest (SER of 1.013). This increased fairness, however, corresponds to lower IoU scores. The least fair models are the stratified model for ResNet18 (SER of 1.047) and the balanced model (SER of 1.155) for EfficientNet-B0.

Looking at the Dice scores across sex groups, shown in Table 3b, we observe similar patterns. The baseline models for ResNet18 and EfficientNet-B0 achieve a Dice score of 0.925 for males 0.923 for females, 0.922 for males, and 0.919 for females. The group-specific models for the ResNet18 backbone exhibit the highest fairness (SER of 1.015), while for EfficientNet-B0 the baseline is the fairest model (SER of 1.036). The least fair models are the stratified model for ResNet18 (SER of 1.055) and the balanced model for EfficientNet-B0 (SER of 1.206). Figures 4c,d and 5c,d illustrate these trade-offs, showing that higher fairness often comes at the cost of lower performance for both IoU and Dice scores.

Lastly, we look at the results for hip segmentation for different age groups, which is presented in Table 2c. For IoU scores, the ResNet18 baseline achieves an IoU of 0.868 for ages 50 or lower, 0.874 for ages 51–64, and 0.871 for ages 65-79, with the least bias (SER of 1.044). For the EfficientNet-B0 baseline, the IoU scores were 0.866, 0.868, and 0.864 for the same age groups, respectively, and are also the least biased (SER of 1.025). The group-specific models were the worst in terms of fairness, with an SER value of 1.574 for ResNet18 and 1.854 for EfficientNet-B0, mainly due to the significant performance differences between the youngest and older age groups. These large differences between the age groups can be seen visually in Figs. 4e and 5e. We see similar patterns with the Dice scores, shown in Table 3c. The ResNet18 baseline had an SER of 1.058, and EfficientNet-B0 had an SER of 1.028. The group-specific models perform the worst in fairness, with much higher SER values, 2.049 for ResNet18 and 2.581 for EfficientNet-B0. The balanced and stratified SER values across both models were very similar to that of IoU. This is illustrated in Figs. 4f and 5f. More investigation into the differences between the age groups is needed.

**Table 4:** IoU and fairness scores for knee segmentation across different protected attributes, including race, sex, and age.

U-Net Backbone	Model	White/Caucasian	Black/AA*	SER	SD	
(a) Racial group IoU & fairness scores						
ResNet18	Baseline	0.924	0.924	1.005	0.000	
	Balanced	0.903	0.920	1.210	0.008	
	Stratified	0.916	0.917	1.008	0.000	
	Group	0.918	0.917	1.008	0.000	
EfficientNet-B0	Baseline	0.924	0.926	1.023	0.001	
	Balanced	0.908	0.923	1.182	0.007	
	Stratified	0.924	0.926	1.024	0.001	
	Group	0.909	0.921	1.159	0.006	

U-Net Backbone	Model	Male	Female	SER	SD	
(b) Sex group IoU & fairness scores						
ResNet18	Baseline	0.924	0.923	1.013	0.000	
	Balanced	0.902	0.909	1.073	0.003	
	Stratified	0.921	0.920	1.017	0.001	
	Group	0.914	0.921	1.087	0.003	
EfficientNet-B0	Baseline	0.926	0.924	1.019	0.001	
	Balanced	0.918	0.911	1.094	0.004	
	Stratified	0.922	0.920	1.033	0.001	
	Group	0.919	0.918	1.010	0.000	

U-Net Backbone	Model	Age 50 or Lower	Age 51–64	Age 65–79	SER	SD
(c) Age group IoU & fairness scores						
ResNet18	Baseline	0.923	0.924	0.924	1.022	0.001
	Balanced	0.912	0.893	0.894	1.215	0.009
	Stratified	0.924	0.927	0.924	1.044	0.001
	Group	0.824	0.911	0.916	2.083	0.042
EfficientNet-B0	Baseline	0.924	0.927	0.922	1.057	0.002
	Balanced	0.910	0.895	0.896	1.174	0.007
	Stratified	0.925	0.927	0.925	1.025	0.001
	Group	0.889	0.919	0.913	1.373	0.013

*AA: African American.

**Table 5:** Dice and Fairness scores for knee segmentation across different protected attributes, including race, sex, and age.

U-Net Backbone	Model	White/Caucasian	Black/AA*	SER	SD	
(a) Racial group dice scores & fairness scores						
ResNet18	Baseline	0.959	0.959	1.004	0.000	
	Balanced	0.946	0.957	1.241	0.005	
	Stratified	0.955	0.955	1.001	0.000	
	Group	0.955	0.956	1.016	0.000	
EfficientNet-B0	Baseline	0.959	0.960	1.024	0.000	
	Balanced	0.949	0.958	1.215	0.004	
	Stratified	0.959	0.960	1.024	0.000	
	Group	0.950	0.958	1.183	0.004	

U-Net Backbone	Model	Male	Female	SER	SD	
(b) Sex group dice score & fairness scores						
ResNet18	Baseline	0.960	0.959	1.010	0.000	
	Balanced	0.947	0.951	1.072	0.002	
	Stratified	0.957	0.957	1.003	0.000	
	Group	0.953	0.957	1.106	0.002	
EfficientNet-B0	Baseline	0.960	0.959	1.002	0.000	
	Balanced	0.956	0.952	1.097	0.002	
	Stratified	0.959	0.957	1.040	0.001	
	Group	0.956	0.956	1.004	0.000	

U-Net Backbone	Model	Age 50 or Lower	Age 51–64	Age 65–79	SER	SD
(c) Age Group Dice Score & Fairness Scores						
ResNet18	Baseline	0.959	0.960	0.959	1.022	0.000
	Balanced	0.953	0.941	0.940	1.269	0.006
	Stratified	0.960	0.961	0.959	1.065	0.001
	Group	0.881	0.951	0.954	2.604	0.034
EfficientNet-B0	Baseline	0.960	0.961	0.957	1.098	0.002
	Balanced	0.952	0.942	0.941	1.215	0.005
	Stratified	0.960	0.961	0.960	1.036	0.001
	Group	0.938	0.957	0.952	1.427	0.008

*AA: African American.

**Figure 6:**
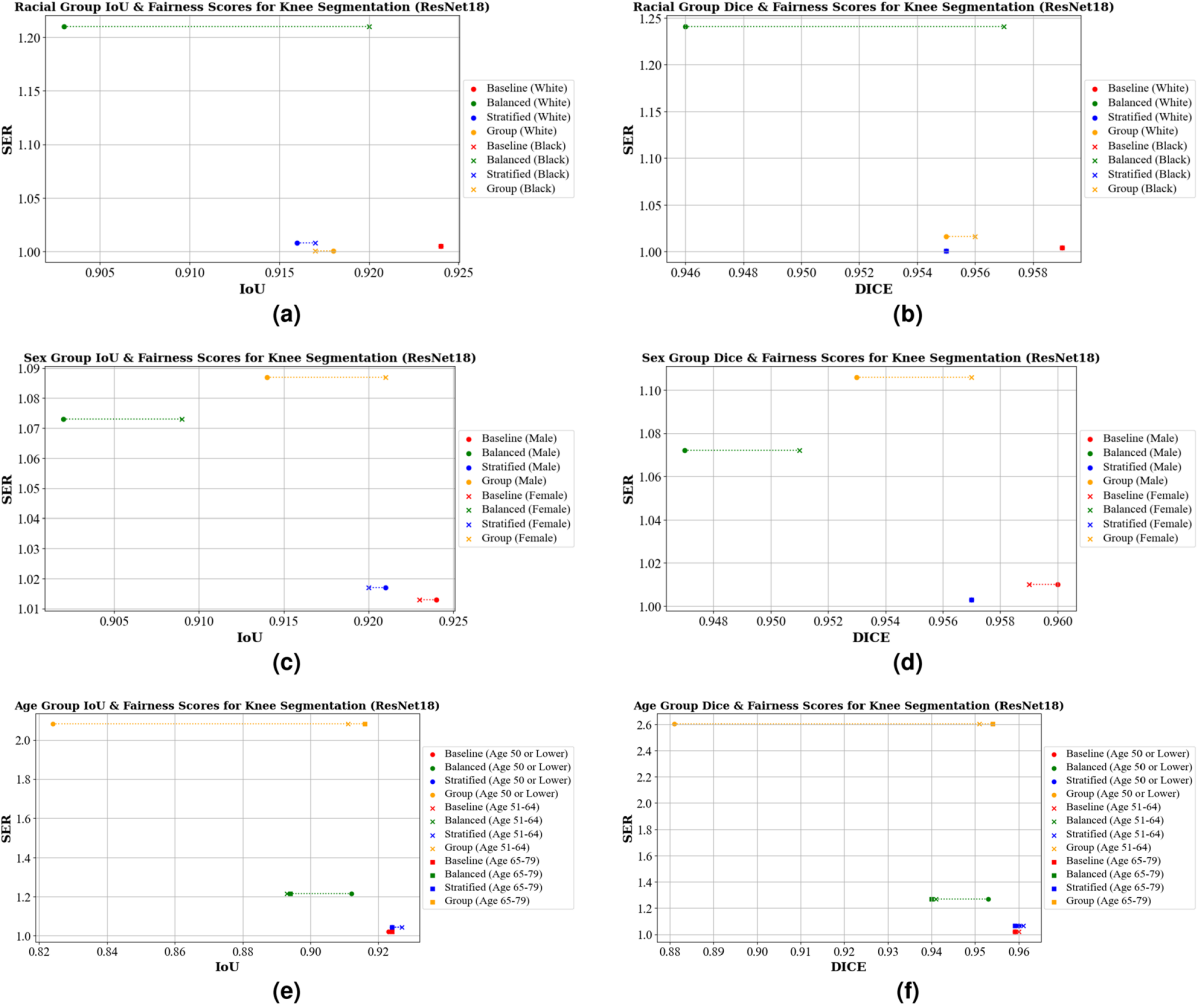
IoU, Dice, and Fairness Scores for knee segmentation using a U-Net model with a ResNet18 backbone. Diagrams (**a**) and (**b**) present the IoU, Dice, and Fairness scores for racial groups. Diagrams (**c**) and (**d**) show the IoU, Dice, and Fairness scores for sex groups. Diagrams (**e**) and (**f**) depict the IoU, Dice, and Fairness scores for age groups.

**Figure 7:**
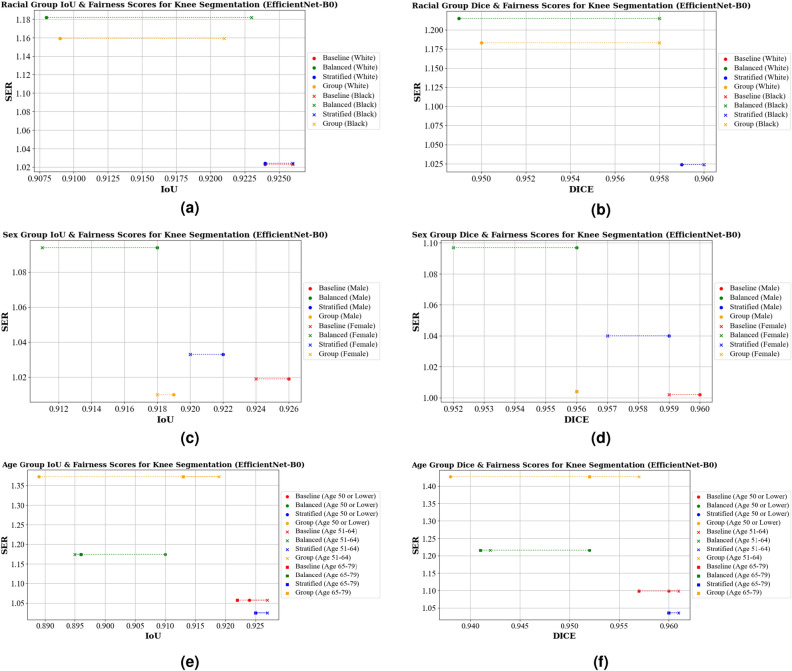
IoU, Dice, and Fairness Scores for knee segmentation using a U-Net model with an EfficientNet-B0 backbone. Diagrams (**a**) and (**b**) present the IoU, Dice, and Fairness scores for racial groups. Diagrams (**c**) and (**d**) show the IoU, Dice, and Fairness scores for sex groups. Diagrams (**e**) and (**f**) depict the IoU, Dice, and Fairness scores for age groups.

Shifting our focus to knee bony anatomy segmentation, Table 4a shows the IoU scores across different racial groups, where we observe noteworthy results. The ResNet18 baseline excels in this context, achieving impressive IoU scores of 0.924 for both White/Caucasian and Black/African American groups. The balanced model is the least fair, with an SER of 1.210, and experiences a significant IoU drop to 0.903 for males, though it maintains 0.920 for females. For EfficentNet-B0 the baseline model was the best, with an IoU of 0.924 and 0.926 for the same groups, respectively, and an SER value of 1.013. The balanced model was the least fair with a SER of 1.182. Figures 6a and 7a visualize these findings.

When looking at the Dice scores, shown in Table 5a for the ResNet18 baseline, stratified, and group-specific models all excelled in delivering high Dice scores for racial groups, but with different SER values (SER of 1.004, 1.001, and 1.016, respectively). The EfficientNet-B0 baseline and stratified models perform equally well, with a Dice score of 0.959 and 0.960 and a SER value of 1.024. Balanced and group-specific models achieve similar Dice scores, but differ in fairness (SER values of 1.215 and 1.183). The similarities between these can be seen visually in Figs. 6b and 7b.

Moving over to looking at sex groups and knee segmentation for IoU, shown in Table 4b, the baseline models for ResNet18 and EfficientNetB0 achieve the best IoU performance. The ResNet18 baseline achieves 0.924 for males and 0.923 for females, with an SER value of 1.013, making it the most fair model compared to the balanced, stratified, and group-specific (SER values of 1.073, 1.017, and 1.087). For EfficientNet-B0, the group based models are the most fair with a SER value of 1.010, but shows a slight decrease in the IoU scores (0.919 for males and 0.918 for females). Stratified and balanced are the least fair, with SER values of 1.033 and 1.094, respectively.

In terms of the dice scores for sex groups, shown in Table 5b, the baseline models again have the best performance, with 0.960 and 0.959 for ResNet18 and EfficientNet-B0 backbones. The most fair models are stratified for ResNet18 (SER of 1.003) and group-based for Efficient-B0 (SER of 1.004). The least fair model for ResNet18 was group-specific models, with an SER value of 1.106). The most fair for EfficientNet-B0 is the baseline model, with a SER value of 1.003. The balanced model is the least fair, with an SER value of 1.097. These trends are visually represented in Figs. 6c,d and 7c,d.

Lastly, we look at the fairness across age groups for knee segmentation. We first look at IoU scores, presented in Table 4c. For the ResNet18 backbone, the baseline model performs the best in terms of both performance and fairness, with IoU scores of 0.923 for age 50 or lower, 0.924 for age 52–64, and 0.924 for age 65–79, and a SER value of 1.022. The group-specific model is the least fair (SER of 2.083), with a significant decrease in IoU for age 50 or lower (0.824). Looking at the EfficientNet-B0 models, the baseline model also had the best performance, with with IoU scores of 0.924, 0.927, and 0.922 for the same age groups, and a SER of 1.057. The group-specific models are the least fair with a SER of 1.373.

For the Dice scores, presented in Table 5c, the best performing and fair models for ResNet18 was the baseline, with scores of 0.959 for age 50 or lower, 0.960 for age 51–64, and 0.959 for ages 65–79, with a SER of 1.022. The group-specific models were the least fair with a SER of 2.604. For the EfficientNet-B0 baseline, the Dice scores are 0.960, 0.961, and 0.957 for the same age groups, respectively, with a SER of 1.098. For this model, the most fair was the stratified model with a SER of 1.065. The most biased models were group-specific, with a SER of 1.427, respectively. These patterns are visually depicted in Figs. 6e,f and 7e,f.

### Significance testing

For protected attributes where the least bias model was not the baseline, t-tests were performed to determine if the resulting SER values were significantly different compared to the baseline. For hip segmentation, this is shown in Table 6, and for the knee, this is shown in Table 7. The null and alternative hypotheses are given below:

6 $$\begin{aligned} H0: SER_{Baseline} = SER_{BestModel} \text { vs. } H1: SER_{Baseline} > SER_{BestModel} \end{aligned}$$

**Table 6:** Significant testing for the SER values of the most fair hip segmentation models for different protected attributes compared to baseline performance.

U-Net backbone	Protected attribute	SER metric	Best model	p-value	Reject
ResNet18	Race	IoU	Group	0.000	Yes
		Dice	Group	0.000	Yes
	Sex	IoU	Balanced	0.745	No
		Dice	Group	0.143	No
EfficientNet-B0	Race	IoU	Balanced	0.871	No
		Dice	Balanced	0.866	No

**Table 7:** Significant testing for the SER values of the most fair knee segmentation models for different protected attributes compared to baseline performance.

U-Net backbone	Protected attribute	SER metric	Best model	p-value	Reject
ResNet18	Race	Dice	Stratified	0.055	No
	Sex	Dice	Stratified	0.707	No
EfficientNet-B0	Race	Dice	Stratified	0.523	No
	Sex	IoU	Group	0.038	Yes
	Age	Dice	Stratified	0.367	No

All testing was done at a significance level of $\alpha =0.05$.

For the hip, most of the fair models were from the ResNet18 backbone. For race, both SER values for IoU and Dice were statistically significant, indicating an improvement in the overall fairness of the model using both metrics. For sex, however, both SER values were not statistically significant, suggesting that while there was an improvement in the overall fairness, it is not significantly different from the baseline model. We see these same results for the race attribute and EfficientNet-B0 backbone. For the knee, we observe strikingly different results. In this case, most of the models that exhibited an increase in fairness were not statistically different from the baseline models. However, this could be attributed to how closely related the models are and minor changes in the overall fairness. The only model statistically different for segmentation was the EfficientNet-B0 backbone for the different sex groups.

## Discussion and outlook

AI-driven image segmentation is a computational strategy that is routinely used in orthopedic surgery. However, fairness becomes crucial when orthopedic surgeons analyze anatomical differences, such as those between the male and female pelvis. The male pelvis resembles the shape of a deep, narrow, inverted cone or a funnel, which can complicate pelvic surgeries in men. Conversely, the female pelvis is more open, wider, and shallower, its elliptical shape adapted to accommodate pregnancy and childbirth, unlike the male pelvis. Furthermore, hip anatomy, such as the angle and orientation of the acetabulum and the femoral neck, may vary among racial groups^42–44^, impacting the risk of hip-related conditions like hip osteoarthritis or hip impingement. Racial disparities in bone density persist even after adjusting for anthropometric, lifestyle, and biochemical factors, with African Americans showing higher bone density levels compared to their White counterparts. Additionally, age-related changes, such as the decline in bone mineral density, vary significantly and are crucial for diagnosing and treating conditions like osteoporosis. Motivated by these anatomical differences and the role of AI-driven image segmentation in orthopedic surgery, this work aimed to build, train, and validate fair and unbiased AI models in autonomous segmentation of knee and hip bony anatomies.

Our study reveals that the pursuit of fairness frequently requires trade-offs that can affect traditional segmentation performance. Different strategies yield varying effects on fairness (SER metric) and segmentation accuracy (IoU or Dice metric) in the context of knee and hip bony anatomy segmentation tasks. The selection of an approach, whether it is the baseline, balanced, stratified, or group-specific, should be guided by the specific segmentation task, such as knee or hip, and the nature of the bias under scrutiny, whether it is racial, age, or sex-related, as well as the encoder backbone being used. While the balanced and group-specific models emerge as the preferred options for hip segmentation, stratified modeling ranks first for knee segmentation in terms of fairness, demonstrating remarkable effectiveness in mitigating racial bias in knee segmentation and achieving nearly perfect results. Likewise, our study reveals that there is always a bias-mitigation trade-off, showing that more often than not when the fairness of the model increases, it is at the cost of the overall performance of the segmentation model. With that, our research findings can be summarized as follows:

- Optimal approach for hip/knee segmentation: The balanced and group-specific modeling approach emerges as the preferred option for hip segmentation, showcasing its effectiveness. Additionally, the balanced modeling also ranks second for knee segmentation, underlining its versatility across anatomical regions. Stratified batching models demonstrate remarkable effectiveness in mitigating racial bias specifically in knee segmentation.
- Selection of an optimal approach: The selection of an approach, whether baseline, balanced, stratified batching or group-specific, as well as the evaluation metric being used, should be informed by the segmentation task at hand (e.g., knee or hip). Our findings underscore the importance of task-specific considerations in the context of deep learning models for knee and hip anatomy segmentation.
- Bias-mitgation tradeoff: When improving the fairness of any of the models there is always the trade-off of losing overall performance in metrics such as IoU and the Dice score for image segmentation. Fairness and accuracy must be carefully managed to maintain both ethical standards and patient care. Minor changes in performance may be acceptable if they lead to better equitable health outcomes. However, it is crucial to prioritize achieving the highest performance at the same time to avoid compromising patient safety and treatment.

Moreover, our research represents a significant advancement in technical and clinical perspectives by developing an automated image segmentation system tailored for knee and hip bony anatomy using plain radiographs. This pioneering effort in AI-driven medical image segmentation, specifically focusing on the intricate bony anatomy of the hip and knee, stands out as the first scholarly attempt to quantify biases within orthopedic deep learning models, particularly those related to sex, race, and age disparities. Through the creation of a gold-standard fully-annotated imaging dataset for fully automatic segmentation, we gained valuable insights into the origins of these biases and proposed comprehensive strategies for ensuring a just, impartial, and safe automatic segmentation process. The system’s impact extends beyond research, playing a vital role in promoting equitable healthcare by offering unbiased knee/hip bony anatomy segmentation in various orthopedic surgery stages. This includes pre-operative planning and personalized surgical plans, enhancing precision, reducing complications, and optimizing long-term success in Total Knee/Hip Arthroplasty (TKA/THA) implant selection. Addressing sex, age, and racial biases actively promotes fair, safe, and equitable care, making a noteworthy contribution to the reduction of health disparities across diverse demographics. Additionally, applications to surgical monitoring intraoperatively are relevant as segmentation tools that are unbiased and accurate would be useful in this clinical setting. While CT and/or MRI is preferred for investigation of more subtle or occult fracture visualizations (e.g., tibial plateau, joint surfaces, growth plate) and are more useful in the setting of trauma, plain radiographs (X-rays) are still widely used for initial assessments and also automated segmentation systems can provide great value at this stage in the longitudinal clinical workup. Our study’s focus on quantifying biases of radiograph segmentation models aims to enhance the clinical utility of this ubiquitous imaging modality, making advanced segmentation techniques more broadly applicable and equitable across diverse healthcare environments.

Our future work will focus on refining bias mitigation strategies in AI-powered medical image segmentation, investigating the development of more advanced and adaptable computational methods to reduce not only sex, race, and age-related biases but also other potential biases related to body size, or comorbidities. There is also a pressing need for more comprehensive and diverse imaging datasets. Future efforts should concentrate on curating imaging datasets that represent a wider spectrum of sex, race, and age diversity to ensure that AI models are effective for a broader and more diverse patient population. Additional attention is needed to conduct longitudinal studies to assess the long-term impact of AI-driven segmentation on patient care, surgical outcomes, and healthcare costs. Furthermore, fostering collaboration among AI researchers, healthcare practitioners, and ethicists is crucial for effectively addressing the ethical considerations associated with AI in healthcare.

## Data Availability

All Python codes and implementation plus a sample dataset are freely and publicly available for any research and educational purposes: https://github.com/pitthexai/AI_Fairness_in_Hip_and_Knee_Bony_Anatomy_Segmentation; The dataset analyzed during the current study is publicly available at the Osteoarthritis Initiative (OAI)^30^.
